# Preventing atrial fibrillation recurrence with combination of catheter ablation and renal denervation or ganglion plexus ablation: A systematic review and network meta-analysis

**DOI:** 10.1016/j.ihj.2025.08.004

**Published:** 2025-09-04

**Authors:** Sebastian Emmanuel Willyanto, Liliana Dewi, Rizki Hari Mulia, Imke Maria Del Rosario Puling, Nyoman Deva Pramana Giri, Derren David Christian Homenta Rampengan, Ardian Rizal

**Affiliations:** a Bachelor Study Program of Medicine, Faculty of Medicine, Universitas Brawijaya, Malang, Indonesia; b Bachelor Study Program of Medicine, Faculty of Medicine, Universitas Sam Ratulangi, Manado, Indonesia; c Brawijaya Cardiovascular Research Center, Department of Cardiology and Vascular Medicine, Faculty of Medicine, Universitas Brawijaya, Malang, Indonesia

**Keywords:** Catheter ablation, Renal denervation, Ganglion plexus ablation, Atrial fibrillation, Recurrence

## Abstract

**Background:**

Atrial fibrillation (AF), affects around 2 % of the global population and is projected to rise over the next 50 years. Catheter ablation (CA) is the primary treatment for symptomatic AF resistant to drug therapy. Despite its widespread use, CA has a failure rate of 20 %–50 %, often requiring repeat procedures, due to significant long-term recurrence rates. Combining CA with renal denervation (RDN) or ganglion plexus ablation (GPA) may effectively reduce the recurrence rates of AF.

**Methods:**

Quality assessment was done using the Cochrane ROB 2.0 tool, network meta-analysis using RStudio, and comparative meta-analysis using RevMan 5.4.

**Results:**

A thorough search across seven databases resulted in 13 articles for analysis, with eight classified as low-risk and five as moderate-risk of bias. The network meta-analysis found that RDN + CA had the highest freedom from AF episodes at 12 and 24 months (OR 2.28 [1.34–3.86] and OR 1.61 [0.89–2.89]), followed by GPA + CA (OR 1.88 [0.91–3.89] and OR 1.36 [0.91–2.03]), compared to CA alone. RDN + CA also showed fewer procedure-related complications (OR 0.78 [0.30–2.02]), while GPA + CA was more prevalent (OR 3.60 [1.72–7.55]), compared to CA alone. Additionally, RDN + CA significantly reduced systolic blood pressure (SBP) (MD -5.22 [-9.91 to −0.53]), diastolic blood pressure (DBP) (MD -3.61 [-7.98 to −0.76]), and creatinine levels (MD -0.25 [-0.34 to −0.15]), while increasing estimated glomerular filtration rate (eGFR) (MD 7.98 [-1.16-17.11]) compared to the control group.

**Conclusion:**

Remarkable success in preventing AF recurrence was observed when CA was combined with RDN or GPA. However, it is noteworthy that GPA + CA was associated with a higher incidence of procedural-related complications, while RDN + CA demonstrated additional advantages by improving blood pressure regulation and renal function.

## 1 Introduction

Atrial fibrillation (AF), characterized by irregular or quivering heartbeats, is a prevalent cardiac arrhythmia that poses significant health risks such as blood clots, stroke, and heart failure.^1^ By 2030, AF is projected to affect over 12 million people, establishing it as the most common clinically significant cardiovascular disorder of the 21st century^2^^.^ The Global Burden of Disease (GBD) study reported that individuals living with AF increased significantly from 33.5 million in 2010 to more than 59 million in 2019^3^^.^ However, many cases remain undiagnosed until symptoms or complications like ischemic stroke arise, suggesting a potentially higher prevalence. AF is associated with heightened morbidity and mortality, placing a considerable burden on healthcare systems. It elevates the risks of myocardial infarction, stroke, heart failure, dementia, cognitive decline, and both non-cardiac and sudden cardiac death^4^^.^

Catheter ablation (CA) is widely regarded as the primary treatment for symptomatic, drug-refractory AF worldwide, offering superior rhythm control compared to medical therapy. Despite significant technological advancements, CA via pulmonary vein isolation (PVI) often fails to achieve satisfactory long-term success rates, necessitating repeat procedures and leading to substantial AF recurrence rates^5^, ^6^, ^7^, ^8^^.^ Combining CA with renal denervation (RDN) or ganglion plexus ablation (GPA) is a promising approach to address these limitations in AF treatment. Previous studies, such as those by Pokushalov et al (2012) and Kiuchi et al (2016), highlight that combining CA with RDN significantly reduces AF recurrence rates and improves blood pressure management in patients with drug-resistant hypertension.^9^^,^^10^ Integrating RDN into CA is technically feasible and does not complicate procedures, suggesting it as a beneficial adjunctive approach to overcome the limitations of CA alone. Moreover, RDN combined with CA offers additional benefits for regulating blood pressure and renal function, making it a potentially advantageous alternative strategy in managing AF beyond the limitations of CA alone.

Additionally, integrating GPA into CA improves AF-free survival in patients with paroxysmal AF without significantly complicating procedures. It is important to note that combining GPA with CA may elevate procedural risks compared to RDN combined with CA. This paper examines the current state of treating AF, focusing on the limitations of primary treatments with CA and highlighting the potential of integrating RDN or GPA with CA as a promising approach to enhance AF management. Our analysis explores the challenges, efficacy, procedural safety, and prospects of integrating RDN or GPA with CA, emphasizing their crucial role in advancing cardiovascular healthcare and optimizing patient outcomes in AF treatment.

## 2 Methods

This meta-analysis was conducted based on the Preferred Reporting Items for Systematic Reviews and Meta-Analyses (PRISMA) statement guidelines.^11^ This study was registered in PROSPERO with registration number CRD42024555231.

## 3 Search strategy

The literature search was carried out on seven databases, namely PubMed, Epistemonikos, EBSCO, Scopus, Cochrane, ScienceDirect, and ProQuest until July 2024. The literature search was carried out with keywords using Boolean operators: (“renal denervation” AND “catheter ablation” AND “atrial fibrillation”).

### 3.1 Study eligibility criteria

Study Inclusion and exclusion criteria were determined before the literature search to make the results specific and homogenous. The inclusion criteria were 1) data available or accessible in the English language, 2) studies that involve patients with AF as their sample, 3) studies that use catheter ablation in combination with renal denervation or ganglion plexus ablation as treatment, and 4) studies that include at least one data to be analyzed in this study, namely: freedom from AF at 12 or 24 months, procedure-related complications, systolic blood pressure (SBP), and estimated glomerular filtration rate (eGFR). The exclusion criteria were 1) non-human sampling studies and 2) irretrievable articles or articles with incompatible language. Using these inclusion and exclusion criteria, four authors independently assessed the eligibility of the papers, and any disagreements were resolved through discussion.

### 3.2 Outcome measures

This study investigates the efficacy and safety of using catheter ablation in combination with renal denervation or ganglion plexus ablation to reduce the recurrence of AF by analyzing outcomes after the procedure. The research evaluates four post-intervention outcomes: freedom from AF at 12 or 24 months, procedure-related complications, systolic blood pressure (SBP), and estimated glomerular filtration rate (eGFR). All results are gathered from each included study based on their accessibility. Regarding freedom from AF, the assessment is based on clinical follow-up and ambulatory ECG, or other methods, as described in the included studies. The authors independently extracted the outcomes from the included papers for quantitative analysis, resolving any disagreements through discussion and collaboration.

### 3.3 Quality assessment

The assessment of bias in the included non-randomized studies was done using the Cochrane ROB 2.0 tool. Following this, the data obtained from the studies were inputted into the "bias" section of a Microsoft Excel 2021 spreadsheet. Subsequently, the spreadsheet was uploaded to the ROBVIS website to visually display the assessment results using the traffic light system^12^.

### 3.4 Statistical analysis

RStudio was utilized to carry out a network meta-analysis and comparative meta-analysis using RevMan 5.4. RStudio was employed to conduct network meta-analyses. Network meta-analysis was chosen to compare the direct and indirect effects of these strategies with a control group, utilizing the "netmeta" package in RStudio 2020 (RStudio Team, 2020). The network graph, created using the "netgraph" function, visually connected and compared all intervention strategies with the control group. The selection between fixed and random effect models was determined by the observed heterogeneity for each outcome, employing the inverse variance model as the statistical tool. Heterogeneity was assessed using I2 statistics, with cutoff criteria of 0 %, 25 %, 50 %, and 75 % indicating insignificant, low, moderate, and high levels of heterogeneity, respectively. Forest plots were employed to summarize the comparisons of pooled strategies for all outcome measures.

## 4 Results

### 4.1 Study selection and identification

**Fig. 1:**
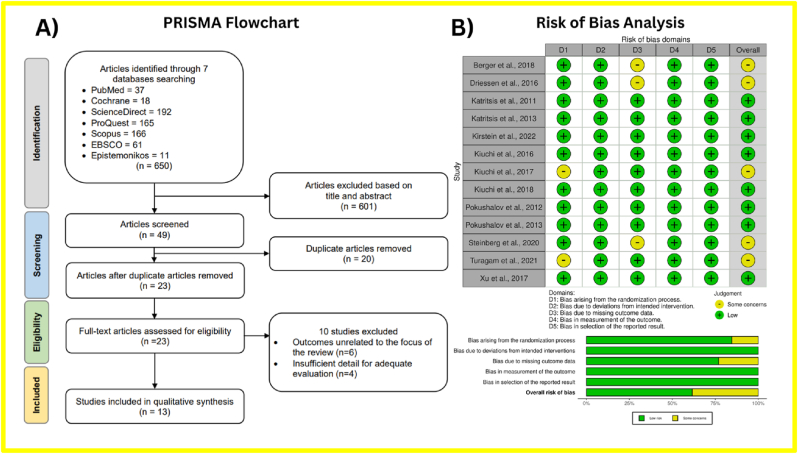
**(A)** Preferred reporting items for systematic reviews and meta-analyses (PRISMA) flowchart for study identification and selection. The original database search resulted in 650 studies from seven databases searched, namely PubMed, Epistemonikos, EBSCO, Scopus, Cochrane, ScienceDirect, and ProQuest. Through title and abstract screening, 601 articles were removed, and 49 articles were screened for duplication. Duplicate screening resulted in 26 removed articles. Twenty-three articles were further assessed for eligibility and ten articles were removed due to irrelevant outcomes and insufficient detail for further analysis. The final step resulted in 13 randomized-controlled trials included in the meta-analysis. **(B)** Risk of bias summary using The Revised Tool for Risk of Bias in Randomized Trials (RoB 2.0) tool for randomized-controlled trials. The green region represents studies with a low risk of bias, the yellow region shows studies with moderate risk of bias, and the red region shows studies with a high risk of bias.

After eliminating duplicate studies and screening abstracts, a detailed assessment was performed on 23 clinical trials. Ultimately, 13 RCTs were included in the meta-analysis, as shown in Fig. 1A. Six studies were excluded because their data were not relevant to the focus of this research, and four were excluded due to insufficient details for a thorough evaluation. The selected studies were assessed for quality and analyzed using statistical methods.

### 4.2 Demography and clinical characteristics of the included studies

The baseline characteristics of each study were reviewed and are detailed in **Supplementary Data 1**. This review showed that the studies were conducted in the Netherlands, Greece, Germany, Brazil, Russia, the USA, and China. Seven studies focused on the efficacy of the combination of CA + GPA in preventing AF, while six studies focused on CA + RDN. The studies on CA + GPA were conducted between 2011 and 2018, whereas the studies on CA + RDN spanned from 2016 to 2022. The primary control was PVI, although one study used PVI combined with 50 mg of spironolactone, and another combined PVI with linear ablation. Additionally, the participants' ages were relatively consistent, with mean ages ranging from the early 50s to late 60s, and the study durations varied from 12 to 55 months. The table provides data on the number of patients with paroxysmal and persistent AF and the CHA2DS2-VASc score for each study. Four studies recorded the AF duration for patients who underwent the intervention, ranging from 4 to 6 years. The mean LVEF data for the included patients ranged from 46 to 65 percent.

### 4.3 Quality appraisal

The RCTs included in the analysis underwent a comprehensive quality evaluation using the RoB 2.0 tool for RCTs from Cochrane. Based on the evaluation of the 12 included studies, it was found that eight studies had a low risk of bias, while four studies had a moderate risk of bias (Fig. 1B). The risk of bias in the randomization process for Turagam et al, 2021 arose because two patients were not randomized due to unfavorable renal anatomy.^13^ The risk of bias in the third domain was due to a significant number of patients lost to follow-up in three studies.^14^, ^15^, ^16^

### 4.4 Freedom from any AF episode within 12 months

**Fig. 2:**
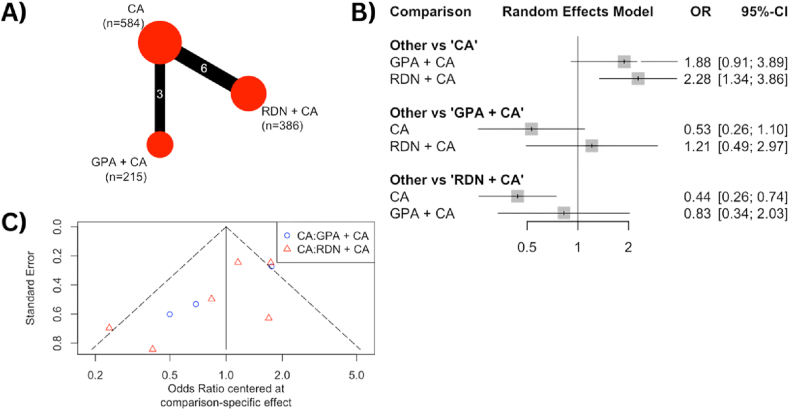
**(A)** The net-graph illustrates both the intervention and control groups, with the size of the red circles representing the number of participants in each respective group. Each connecting line between interventions represents the interconnection and networking status of the particular approach. **(B)** Forest plot of the odds ratio (OR) of freedom from any atrial fibrillation episode within 12 months comparing the combination of catheter ablation with either ganglion plexus ablation or renal denervation and catheter ablation alone as control. The gray square and solid lines represent the mean difference with 95 % confidence intervals. The size of the squares indicates the weight of each study. **(C)** Funnel plot of the freedom from any atrial fibrillation episode within 12 months as a graphical representation of the size of trials plotted against the effect size they report. As the trial size grows, it becomes more probable for trials to converge toward the true underlying effect size. The vertical axis represents the study size, while the horizontal scale represents the effect estimates. Asymmetry in funnel plots may be the result of publication bias.

Nine RCTs reported the outcome of freedom from any AF episode within 12 months and were analyzed using network meta-analysis. The results, shown in Fig. 2A, present a network outcome for RDN + CA involving 386 patients and GPA + CA involving 215 patients as the intervention groups, while 584 patients receiving CA alone served as the control group. The forest plot in Fig. 2B compares the combination of CA with either RDN or GPA against CA alone. Among the intervention groups, the RDN + CA group showed a higher rate of freedom from any AF episode within 12 months (OR 2.28 [1.34–3.86]), whereas the GPA + CA group had an OR of 1.88 [0.91–3.89]. Nonetheless, both combination interventions demonstrated better freedom from any AF episode within 12 months compared to CA alone. The funnel plot in Fig. 2C indicates significant heterogeneity among the studies, as evidenced by its dispersed outcomes.

### 4.5 Freedom from any AF episode within 24 months

**Fig. 3:**
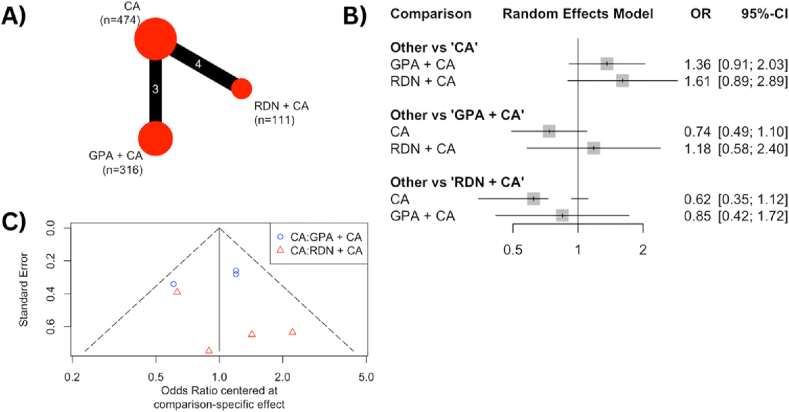
**(A)** The net-graph illustrates both the intervention and control groups, with the size of the red circles representing the number of participants in each respective group. Each connecting line between interventions represents the interconnection and networking status of the particular approach. **(B)** Forest plot of the odds ratio (OR) of freedom from any atrial fibrillation episode within 24 months comparing the combination of catheter ablation with either ganglion plexus ablation or renal denervation and catheter ablation alone as control. The gray square and solid lines represent the mean difference with 95 % confidence intervals. The size of the squares indicates the weight of each study. **(C)** Funnel plot of the freedom from any atrial fibrillation episode within 24 months as a graphical representation of the size of trials plotted against the effect size they report. As the trial size grows, it becomes more probable for trials to converge toward the true underlying effect size. The vertical axis represents the study size, while the horizontal scale represents the effect estimates. Asymmetry in funnel plots may be the result of publication bias.

Seven RCTs reported the outcome of freedom from any AF episode within 24 months and underwent analysis through a network meta-analysis. Fig. 3A shows a network outcome for RDN + CA involving 111 patients and GPA + CA involving 316 patients as the intervention groups, with 474 patients receiving CA alone as the control group. The forest plot in Fig. 3B compares the combinations of CA with either RDN or GPA against CA alone.The RDN + CA group also had a higher rate of freedom from any AF episode within 24 months (OR 1.61 [0.89–2.89]), while the GPA + CA group had an OR of 1.36 [0.91–2.03]. Both combination interventions showed better outcomes for freedom from any AF episode within 24 months compared to CA alone. The funnel plot in Fig. 3C indicates the significant heterogeneity among the studies, showing dispersed outcomes.

### 4.6 Procedural-related complications rate

**Fig. 4:**
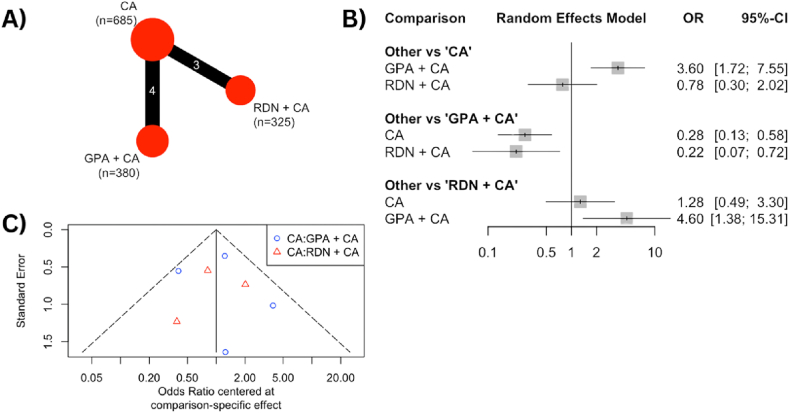
**(A)** The net-graph illustrates both the intervention and control groups, with the size of the red circles representing the number of participants in each respective group. Each connecting line between interventions represents the interconnection and networking status of the particular approach. **(B)** Forest plot of the odds ratio (OR) of procedural-related complications rate comparing the combination of catheter ablation with either ganglion plexus ablation or renal denervation and catheter ablation alone as control. The gray square and solid lines represent the mean difference with 95 % confidence intervals. The size of the squares indicates the weight of each study. **(C)** Funnel plot of the procedural-related complications rate as a graphical representation of the size of trials plotted against the effect size they report. As the trial size grows, it becomes more probable for trials to converge toward the true underlying effect size. The vertical axis represents the study size, while the horizontal scale represents the effect estimates. Asymmetry in funnel plots may be the result of publication bias.

Seven RCTs reported the rate of procedure-related complications and were analyzed through network meta-analysis. The findings, illustrated in Fig. 4A, depict a network outcome for RDN + CA involving 325 patients and GPA + CA involving 380 patients as the intervention groups, with 685 patients receiving CA alone as the control group. The forest plot in Fig. 4B compares the combination of CA with either RDN or GPA against CA alone. The RDN + CA group demonstrated a lower complication rate compared to the control group (OR 0.78 [0.30–2.02]), whereas the GPA + CA group exhibited a higher complication rate compared to the CA alone group (OR 3.60 [1.72–7.55]). The funnel plot in Fig. 4C shows significant heterogeneity among the studies, as evidenced by its dispersed outcome.

### 4.7 Specific outcomes for the combination of renal denervation and catheter ablation

#### 4.7.1 Systolic blood pressure mean change in 12 months

**Fig. 5:**
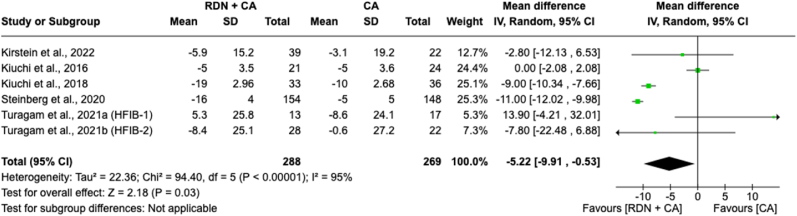
Forest plot of the mean difference of systolic blood pressure mean change in 12 months between individuals with the combination of renal denervation and catheter ablation alone. The green square and solid lines represent the mean difference with 95 % confidence intervals. The size of the squares indicates the weight of each study. The black rhombus indicates the pooled estimate with 95 % confidence intervals.

Meta-analysis of the mean change in SBP over 12 months between individuals receiving RDN + CA versus CA alone involved 557 patients across five included studies (Fig. 5). The forest plot indicated a significant overall effect, showing a reduction in SBP with an MD of −5.22 [−9.91 to −0.53]; *p* = 0.03) compared to CA alone. However, there is considerable heterogeneity (*I*^*2*^ = 95 %) among the study effect sizes, indicating significant variability in the pooled result.

#### 4.7.2 Diastolic blood pressure mean change in 12 months

**Fig. 6:**
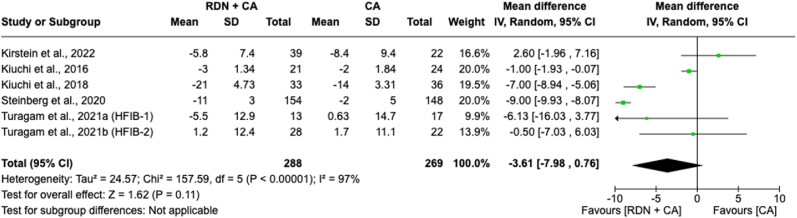
Forest plot of the mean difference of systolic blood pressure mean change in 12 months between individuals with the combination of renal denervation and catheter ablation alone. The green square and solid lines represent the mean difference with 95 % confidence intervals. The size of the squares indicates the weight of each study. The black rhombus indicates the pooled estimate with 95 % confidence intervals.

A meta-analysis of the mean change in DBP over 12 months between individuals receiving RDN + CA versus CA alone included 557 patients across five included studies (Fig. 6). The forest plot revealed a non-significant overall effect, with a reduction in DBP showing an MD of −3.61 [−7.98-0.76]; *p* = 0.11) compared to CA alone. However, there was notable heterogeneity (*I*^*2*^ = 97 %) among the study effect sizes, indicating significant variability in the pooled results.

#### 4.7.3 Estimated glomerular filtration rate mean change in 12 months

**Fig. 7:**
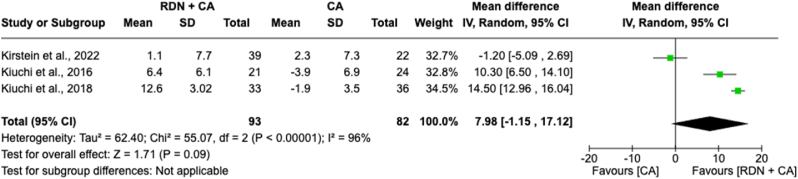
Forest plot of the mean difference of estimated glomerular filtration rate mean change in 12 months between individuals with the combination of renal denervation and catheter ablation alone. The green square and solid lines represent the mean difference with 95 % confidence intervals. The size of the squares indicates the weight of each study. The black rhombus indicates the pooled estimate with 95 % confidence intervals.

A meta-analysis of the mean change in eGFR over 12 months between individuals receiving RDN + CA versus CA alone included 175 patients across three studies (Fig. 7). The forest plot revealed a non-significant overall effect, showing an increase in eGFR with an MD of 7.98 [−1.15-17.12]; *p* = 0.09) compared to CA alone. However, there was notable heterogeneity (*I*^*2*^ = 96 %) among the study effect sizes, indicating significant variability in the pooled results.

#### 4.7.4 Creatinine levels mean change in 12 months

**Fig. 8:**
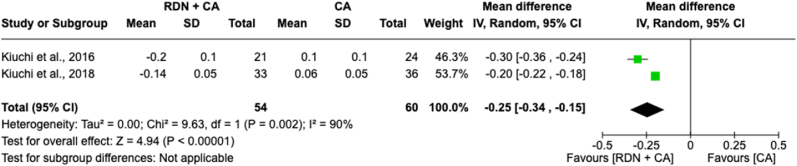
Forest plot of the mean difference of creatinine levels mean change in 12 months between individuals with the combination of renal denervation and catheter ablation alone. The green square and solid lines represent the mean difference with 95 % confidence intervals. The size of the squares indicates the weight of each study. The black rhombus indicates the pooled estimate with 95 % confidence intervals.

Meta-analysis of creatinine levels mean change in 12 months between individuals with RDN + CA and CA alone intervention involved 114 patients across two included studies (Fig. 8). The forest plot revealed a significant overall effect, with a reduction of creatinine levels with an MD of −0.25 [−0.34 to −0.15]; *p* < 0.00001) favoring the combination of RDN compared to CA alone. However, there is notable heterogeneity (*I*^*2*^ = 90 %) among the effect sizes of the studies, which accounts for the significant variability in the pooled result.

## 5 Discussion

Catheter ablation for AF has become a key rhythm-control technique and is the most frequently performed cardiac ablation procedure globally. According to current guidelines, the procedure is recommended for symptomatic patients with paroxysmal or persistent AF who do not respond to or cannot tolerate antiarrhythmic drugs. It may also be considered as a first-line treatment for certain asymptomatic patients. The primary technique for AF ablation is PVI, which can be done using point-by-point radiofrequency or a cryoballoon. While additional atrial ablation can be carried out in patients with persistent AF, the benefits of this approach are not well-established.^17^^,^^18^

RDN is a minimally invasive procedure that employs a catheter to deliver radiofrequency, ultrasound, or alcohol to disrupt the nerve signals between the kidney and brain by targeting the renal afferent and efferent nerves.^19^^,^^20^ The indications for RDN are outlined in the 2023 European Society of Hypertension (ESH) guideline^21^^,^ the 2022 Taiwan Hypertension Society (THS) guideline^22^^,^ and the 2021 Society for Cardiovascular Angiography and Interventions (SCAI) consensus^23^^,^ which specify that RDN is recommended for patients with (1) Refractory, resistant, or uncontrolled hypertension (defined as BP exceeding 140/90 mmHg) despite treatment with at least three antihypertensive medications, including a diuretic at optimal doses; (2) Exclusion of secondary hypertension and white coat hypertension through ambulatory blood pressure monitoring; (3) Intolerance to medications due to serious side effects or a significantly reduced quality of life; (4) features suggesting neurogenic hypertension after thorough clinical and imaging assessment; and (5) An estimated glomerular filtration rate (GFR) over 40 ml/min/1.73 m^2^. Although not yet included in guidelines, two previous meta-analyses^24^^,^^25^^,^ suggest that combining RDN with CA in hypertensive patients with AF is more effective and superior to using CA alone for treating AF.

GPA is a long-term catheter-based procedure designed to disrupt the neural pathways that regulate heart rhythm by targeting and ablating clusters of autonomic ganglia on the heart's epicardial surface, with the majority embedded in epicardial fat pads.^26^^,^^27^ GPA for the treatment of AF is not a new technique; it has been a controversial topic and is not recommended in current established guidelines because of the lack of a sensitive and specific means to localize the GP in patients^28^^.^ However, a case report describes the first instance of GPA in humans, where solely targeting the GP-triggering AF resulted in long-term freedom from the condition. This was achieved by performing synchronized high-frequency stimulation (HFS) to specifically identify the particular type of GP^29^. Although not superior to PVI on its own, GPA has potential when combined with PVI, showing improved success rates and reduced AF burden. Evidence suggests that combining GPA with traditional PVI yields a 74 % success rate in treating paroxysmal AF, significantly higher than the 56 % success rate for PVI alone at 12 months, whereas GPA without PVI results in a lower success rate of 48 %.^20^

GPA enhances the effectiveness of PVI by targeting neural pathways contributing to AF, resulting in better pulmonary vein isolation and a lower incidence of spontaneous pulmonary vein firing.^30^, ^31^ This procedure can also reduce the AF burden by modulating the autonomic nervous system, leading to fewer and shorter AF episodes.^32^ A meta-analysis of RCTs comparing GPA combined with PVI to PVI alone found that adding GPA significantly reduced arrhythmia recurrence in patients with paroxysmal AF, suggesting it may be a more effective treatment.^33^ Another network meta-analysis of RCTs comparing different CA strategies for paroxysmal AF indicated that PVI combined with additional ablation techniques had significantly higher freedom from arrhythmia compared to PVI alone. These findings suggest that combining PVI with GPA or other adjuvant techniques may offer enhanced effectiveness over PVI alone.^34^

The combination of PVI with RDN has shown remarkable results. In our network meta-analysis assessment, the PVI + RDN group showed a higher OR of freedom from AF within 12 and 24 months indicating the success of PVI + RDN in reducing AF recurrence. While showing remarkable efficacy, PVI + RDN also showed outstanding safety by showing fewer procedural-related complications compared to PVI + GPA and PVI alone groups. Moreover, beyond the impressive results from the network meta-analysis, the combination of PVI + RDN was found to significantly lower systolic and diastolic BP and reduce creatinine levels. This therapy also increases the eGFR in patients suffering from resistant hypertension, offering a comprehensive benefit in managing cardiovascular health. The efficacy and safety profile of the PVI + RDN combination underscores its potential as a superior treatment option for patients with AF and resistant hypertension, providing both symptomatic relief and improved clinical outcomes.

The incremental benefit of RDN in rhythm control likely stems from its modulation of the sympathetic nervous system. Heightened sympathetic tone contributes to AF initiation and perpetuation by promoting triggered activity and structural remodeling of atrial tissue. RDN reduces renal sympathetic afferent and efferent activity, thereby decreasing central sympathetic outflow.^19^^,^^20^ This modulation may stabilize atrial electrophysiology and reduce arrhythmogenic substrates—particularly important in persistent AF, where structural and autonomic remodeling is more advanced. By lowering systemic blood pressure and sympathetic drive, RDN may also reduce atrial stretch and remodeling.^35^, ^36^

Our findings are consistent with prior meta-analyses. Nawar et al (2022), including 711 patients from seven studies, found that RDN + CA reduced AF recurrence (31.3 %) compared to CA alone (52.9 %), and significantly decreased systolic blood pressure by 9.42 mmHg while increasing eGFR by 10.2 mL/min/1.73 m^2^.^24^

Similarly, Pranata et al (2020) reported that RDN + CA/PVI was associated with reduced AF recurrence (HR 0.51) and a similar complication rate to CA alone (RR = 0.87).^25^ Ukena et al (2020) also observed a significantly lower AF recurrence rate in the PVI + RDN group (OR 0.43), alongside notable BP reductions.^26^

Despite these strengths, several primary studies have reported limitations. The AFFORD study,^37^ which followed 20 patients over three years, showed unchanged AF burden and antiarrhythmic drug use. The ERADICATE-AF trial^16^^,^ reported similar procedural complication rates in both CA-only and RDN + CA groups (∼4.5 %). Additionally, the RDN + AF study^38^^,^ found no significant difference in rhythm outcomes and noted a 15 % rate of moderate periprocedural complications. These findings emphasize the need for cautious patient selection and long-term data, especially in persistent AF.

Our investigation also supports the efficacy of GPA + CA. Network meta-analysis showed a significant improvement in freedom from AF episodes at 12 months (OR 1.88) and sustained benefit at 24 months (OR 1.36) compared to CA alone. The ganglionated plexi are key modulators of cardiac autonomic input, particularly parasympathetic tone. GPA targets these epicardial neural clusters, which are thought to contribute to AF initiation and maintenance. Ablation of GPs during CA can reduce parasympathetic triggers and improve rhythm stability. This may be especially relevant in persistent AF, where autonomic remodeling is more extensive. While GPA + CA improves efficacy, it is associated with a higher complication rate compared to CA alone (OR 3.60). Nonetheless, these risks may be justifiable in selected patients, particularly those with persistent AF, who have historically lower success rates with ablation alone.

However, it is important to note that the GPA techniques employed across the included studies were heterogeneous. Two studies utilized high-frequency stimulation (HFS)-guided GPA to identify and ablate active autonomic ganglia, while two others applied purely anatomic GPA without functional testing. Additionally, two studies employed epicardial GPA during thoracoscopic surgery using a combination of anatomical landmarks and HFS. This variation in ablation strategies may contribute to heterogeneity in treatment effects and should be considered when interpreting the results. Subgroup analysis by GPA technique was not feasible due to the limited number of studies in each subgroup.

Supporting studies align with our findings. Charitakis et al (2022) demonstrated that GPA + CA reduces AF recurrence risk (RR 0.62) but extends procedural time.^34^ Zhou et al (2011) confirmed GPA + CA improved AF freedom at 3–12 months post-ablation without antiarrhythmic drugs.^39^ Kampaktsis et al (2017), across four RCTs with 718 patients, found GPA + CA improved freedom from AT/AF, particularly in paroxysmal AF (75.8 % vs. 60.0 %) and showed a favorable trend in persistent AF.^40^ Although procedure durations and energy delivery times increased, no procedure-related deaths were reported.

While the current data suggest RDN and GPA may offer benefits in persistent AF, this subgroup remains underrepresented in trials. Persistent AF presents more profound structural and autonomic remodeling, rendering standard CA less effective. The mechanisms of RDN and GPA—modulating both sympathetic and parasympathetic inputs—may offer synergistic effects in this population. However, definitive evidence remains limited, and further subgroup analyses and trials focused on persistent AF are needed.

While previous research has primarily focused on single ablation strategies, this study provides a comprehensive evaluation of combination treatments, incorporating data from multiple regions—including Asia, America, and Europe—thus enhancing the generalizability of the findings. However, several limitations must be acknowledged. Clinical heterogeneity among the included studies may affect indirect comparisons. RDN trials predominantly enrolled hypertensive patients, often with chronic kidney disease, whereas GPA trials involved normotensive individuals with paroxysmal AF. Although this reflects real-world variation in AF populations, it may introduce residual confounding. We assessed transitivity using available baseline characteristics (e.g., age, AF type, CHA_2_DS_2_-VASc score), but other important variables such as blood pressure, eGFR, and left atrial size were not consistently reported.

Methodological differences also limit comparability across trials, including variability in AF subtype classification (paroxysmal vs. persistent), imbalance in sex distribution, differences in ablation protocols (e.g., power settings, catheter technologies), and insufficient procedural reporting. Additionally, potential exclusion of non-English publications and studies published after July 2024 may have led to missed relevant evidence. Two reports from the AFACT trial were included as separate entries in this analysis, reflecting distinct follow-up durations at 12 and 24 months. Although this approach allowed us to capture potential temporal variations in treatment effect, we acknowledge the shared patient population and recognize this as a methodological limitation that may introduce partial duplication within the network.

Notably, several recent trials and expert statements—such as SPYRAL-AF (Falkensammer et al, 2025), GANGLIA-AF, CryoDENERATE, the 2024 EHRA/10.13039/100005859HRS consensus, and a meta-analysis comparing cryoablation and RF (Europace, 2024)—were not included but further support the relevance of autonomic modulation in AF therapy. These limitations underscore the need for future well-designed studies with standardized protocols and broader populations to refine the role of autonomic modulation and guide optimal treatment strategies.

## 6 Conclusion

A significant reduction in the recurrence of AF was seen when CA was paired with either RDN or GPA. Nonetheless, it is important to highlight that the combination of GPA and CA was associated to a greater rate of complications related to the procedure. On the other hand, the pairing of RDN with CA offered additional benefits, notably by enhancing blood pressure control and improving renal function. To better understand the role of RDN, further research is required, particularly focusing on its application in specific clinical scenarios and in patients with other comorbidities.

## Patient consent statement

N/A.

## Data availability statement

Data available within the article or its supplementary materials.

## Ethics approval statement

The authors confirm that the ethical policies of the journal, as noted on the journal's author guidelines page, have been adhered to. No ethical approval was required as this is a network meta-analysis and systematic review with no original research data.

## Author contributions

Conceptualization: SEW, LD, RHM, IMDRP, and NDPG. Data collection: SEW, LD, and RHM. Data analysis: SEW and LD. Data curation: SEW, LD, IMDRP, and NDPG. Writing - Original draft: SEW, LD, RHM, IMDRP, NDPG, and DDCHR. Writing - Review and editing: AR. Supervision: AR.

## Clinical trial registration

This study was registered in PROSPERO with registration number **CRD42024555231**.

## Funding STATEMENT

No funding was received.

## Declaration of competing interest

The authors declare that they have no known competing financial interests or personal relationships that could have appeared to influence the work reported in this paper.
