# Supplementary material for: Preventing atrial fibrillation recurrence with combination of catheter ablation and renal denervation or ganglion plexus ablation: A systematic review and network meta-analysis
Source: Indian Heart J. 2025 Sep 4;77(6):439–47. doi: 10.1016/j.ihj.2025.08.004 (PMC12793911; doi:10.1016/j.ihj.2025.08.004)
Supplement: Multimedia component 1 [file mmc1.docx]

#

# **SUPPLEMENTARY DATA**

**Supplementary Data 1.** Demography and clinical characteristics of included studies

| **No.** | **Author, Year** | **Study Design** | **Country** | **Center** | **Duration (months)** | **Type of Intervention** | **Control** | **Age**  **Mean ± SD/Median (years)** | | **Total Patients (n)** | **Sample Size (n)** | | **Sample Size (M/F)** | | **Paroxysmal AF (n)** | | **Persistent AF (n)** | | **CHA2DS2-VASc** | | **AF duration (years)** | | **LVEF (%)** | |
| --- | --- | --- | --- | --- | --- | --- | --- | --- | --- | --- | --- | --- | --- | --- | --- | --- | --- | --- | --- | --- | --- | --- | --- | --- |
|  |  |  |  |  |  |  |  | **Intervention** | **Control** |  | **Intervention** | **Control** | **Intervention** | **Control** | **Intervention** | **Control** | **Intervention** | **Control** | **Intervention** | **Control** | **Intervention** | **Control** | **Intervention** | **Control** |
| 1 | Berger et al., 2018 | RCT | Netherlands | Single center | 24 | GPA + PVI | PVI only | 59.5 ± 8.2 | 60.2 ± 8.2 | 240 | 117 | 123 | 84/33 | 91/32 | 41 | 56 | 76 | 67 | 1.4 ± 1.2 | 1.4 ± 1.3 | 4 (2-6) | 5 (2-10) | 47.9 ± 18 | 51.2 ± 9.1 |
| 2 | Driessen et al., 2016 | RCT | Netherlands | Single center | 12 | GPA + PVI | PVI only | 59.5 ± 8.2 | 60.2 ± 8.2 | 240 | 117 | 123 | 84/33 | 91/32 | 43 | 55 | 74 | 68 | 1.4 ± 1.2 | 1.4 ± 1.3 | 4 (2-6) | 5 (2-10) | 47.9 ± 18 | 51.2 ± 9.1 |
| 3 | Katritsis et al., 2011 | RCT | Greece | Multi center | 12 | GPA + PVI | PVI only | 55.2 ± 11.6 | 53.2 ± 11.3 | 67 | 34 | 33 | 25/9 | 26/7 | NI | NI | NI | NI | NI | NI | NI | NI | 56.2 ± 7.7 | 56.1 ± 5.3 |
| 4 | Katritsis et al., 2013 | RCT | Greece | Multi center | 24 | GPA + PVI | PVI only | 56 ± 8.5 | 56 ± 7.6 | 260 | 82 | 78 | 57/25 | 53/25 | NI | NI | NI | NI | NI | NI | NI | NI | 62 ± 8.1 | 63 ± 6.8 |
| 5 | Kirstein et al., 2022 | RCT | Germany | Multi center | 27 | RDN + CA | CA only | 66.3 ± 7.9 | 63.0 ± 9.9 | 61 | 39 | 22 | 20/19 | 12/10 | 18 | 12 | 21 | 8 | NI | NI | NI | NI | 58.2 ± 9.5 | 58.8 ± 8.9 |
| 6 | Kiuchi et al., 2016 | RCT | Brazil | Single center | 12 | RDN + PVI | PVI only | 68 ± 9 | 66 ± 9 | 45 | 21 | 24 | 13/8 | 16/8 | 12 | 15 | 9 | 9 | 4.7 ± 1.7 | 3.7 ± 1.3 | NI | NI | 62.7 ± 6.6 | 63.5 ± 6.8 |
| 7 | Kiuchi et al., 2018 | RCT | Brazil | Single center | 12 | RDN + PVI | PVI + Spironolactone 50mg | 56.8 ± 6.5 | 58.4 ± 5.1 | 69 | 33 | 36 | 25/8 | 30/6 | 33 | 36 | NI | NI | NI | NI | NI | NI | 62.2 ± 7.2 | 61.2 ± 5.7 |
| 8 | Pokushalov et al., 2012 | RCT | Russia | Single center | 12 | RDN + PVI | PVI only | 57 ± 8 | 56 ± 9 | 27 | 13 | 14 | 11/2 | 10/4 | 4 | 5 | 9 | 9 | NI | NI | 5.7 ± 4.9 | 5.3 ± 3.2 | 65 ± 5 | 66 ± 4 |
| 9 | Pokushalov et al., 2013 | RCT | Russia | Multi center | 36 | GPA + PVI | PVI + Linear Ablation | 55 ± 6 | 54 ± 7 | 264 | 132 | 132 | 101/31 | 105/27 | NI | NI | 89 | 92 | NI | NI | 5.9 ± 3.9 | 5.4 ± 3.6 | 55.1 ± 4.8 | 54.2 ± 6.3 |
| 10 | Steinberg et al., 2020 | RCT | Russia | Multi center | 55 | RDN + PVI | PVI only | 59 (54-65) | 60 (58-65) | 302 | 154 | 148 | 91/63 | 91/57 | NI | NI | NI | NI | NI | NI | NI | NI | 62 ± 5 | 62 ± 5 |
| 11 | Turagam et al., 2021a (HFIB-1) | RCT | USA | Multi center | 52 | RDN + CA | CA only | 59 ± 10 | 68 ± 9 | 30 | 13 | 17 | 8/5 | 8/9 | 8 | 12 | 5 | 5 | NI | NI | NI | NI | 51 ± 0.9 | 46 ± 0.7 |
| 12 | Turagam et al., 2021b (HFIB-2) | RCT | USA | Multi center | 55 | RDN + CA | CA only | 64 ± 7 | 65 ± 8 | 50 | 28 | 22 | 16/12 | 14/8 | 20 | 15 | 8 | 7 | NI | NI | NI | NI | 54 ± 0.9 | 47 ± 1.3 |
| 13 | Xu et al., 2017 | RCT | China | Multi center | 12 | GPA + PVI | PVI only | 59.91 ± 9.5 | 60.51 ± 9.6 | 123 | 64 | 59 | 46/18 | 36/23 | NI | NI | NI | NI | 1.63 ± 1.3 | 1.95 ± 1.3 | NI | NI | NI | NI |

**Supplementary Data 2.** GRADE Summary of Findings Table

| **No.** | **Outcome** | **No. of Participants (Studies)** | **Effect Estimate** | **Certainty of Evidence** | **Reasons for Downgrade** |
| --- | --- | --- | --- | --- | --- |
| 1 | Freedom from AF (12 months) | 1,185 (9 RCTs) | OR 2.28 (95% CI: 1.34 to 3.86) | ⬤⬤⬤◯ Moderate | Risk of bias in 2 studies, inconsistency |
| 2 | Freedom from AF (24 months) | 901 (7 RCTs) | OR 1.61 (95% CI: 0.89 to 2.89) | ⬤⬤◯◯ Low | Imprecision (CI overlaps 1), risk of bias |
| 3 | Procedure-related complications (GPA) | 1,390 (7 RCTs) | OR 3.60 (95% CI: 1.72 to 7.55) | ⬤⬤⬤◯ Moderate | Heterogeneity across GPA techniques |
| 4 | SBP reduction (RDN) | 557 (5 RCTs) | MD -5.22 mmHg (95% CI: -9.91 to -0.53) | ⬤⬤◯◯ Low | High heterogeneity (I² = 95%) |
| 5 | DBP reduction (RDN) | 557 (5 RCTs) | MD -3.61 mmHg (95% CI: -7.98 to -0.76) | ⬤⬤◯◯ Low | High heterogeneity (I² = 97%) |
| 6 | eGFR increase (RDN) | 175 (3 RCTs) | MD +7.98 mL/min/1.73m² (95% CI: -1.16 to 17.11) | ⬤⬤◯◯ Low | Imprecision and heterogeneity (I² = 96%) |
| 7 | Creatinine reduction (RDN) | 114 (2 RCTs) | MD -0.25 mg/dL (95% CI: -0.34 to -0.15) | ⬤⬤⬤◯ Moderate | Small sample size |
